# A scoping review of strategies for financing the implementation of evidence-based practices in behavioral health systems: State of the literature and future directions

**DOI:** 10.1177/2633489520939980

**Published:** 2020-08-30

**Authors:** Alex R Dopp, Marie-Rachelle Narcisse, Peter Mundey, Jane F Silovsky, Allison B Smith, David Mandell, Beverly W Funderburk, Byron J Powell, Susan Schmidt, Daniel Edwards, Douglas Luke, Peter Mendel

**Affiliations:** 1 Department of Behavioral and Policy Sciences, RAND Corporation, Santa Monica, CA, USA; 2 Department of Community Health and Research, University of Arkansas for Medical Sciences, Fayetteville, AR, USA; 3 Department of Social and Behavioral Sciences, Savannah State University, Savannah, GA, USA; 4 Department of Pediatrics, University of Oklahoma Health Sciences Center, Oklahoma City, OK, USA; 5 Department of Psychological Science, University of Arkansas, Fayetteville, AR, USA; 6 Department of Psychiatry, University of Pennsylvania, Philadelphia, PA, USA; 7 Brown School, Washington University in St. Louis, Saint Louis, MO, USA; 8 Evidence-Based Associates, Alexandria, VA, USA; 9 Department of Economics, Sociology, and Statistics, RAND Corporation, Santa Monica, CA, USA

**Keywords:** Financing strategies, evidence-based practice, behavioral health systems, implementation, sustainment

## Abstract

**Background:**

Increased availability of evidence-based practices (EBPs) is essential to alleviating the negative public health and societal effects of behavioral health problems. A major challenge to implementing and sustaining EBPs broadly is the limited and fragmented nature of available funding.

**Method:**

We conducted a scoping review that assessed the current state of evidence on EBP financing strategies for behavioral health based on recent literature (i.e., post-Affordable Care Act). We defined financing strategies as techniques that secure and direct financial resources to support EBP implementation. This article introduces a conceptualization of financing strategies and then presents a compilation of identified strategies, following established reporting guidelines for the implementation strategies. We also describe the reported level of use for each financing strategy in the research literature.

**Results:**

Of 23 financing strategies, 13 were reported as being used within behavioral health services, 4 had potential for use, 5 had conceptual use only, and 1 was potentially contraindicated. Examples of strategies reported being used include increased fee-for-service reimbursement, grants, cost sharing, and pay-for-success contracts. No strategies had been evaluated in ways that allowed for strong conclusions about their impact on EBP implementation outcomes.

**Conclusion:**

The existing literature on EBP financing strategies in behavioral health raises far more questions than answers. Therefore, we propose a research agenda that will help better understand these financing strategies. We also discuss the implications of our findings for behavioral health professionals, system leaders, and policymakers who want to develop robust, sustainable financing for EBP implementation in behavioral health systems.

**Plain language abstract:**

Organizations that treat behavioral health problems (mental health and substance use) often seek to adopt and use evidence-based practices (EBPs). A challenge to adopting EBPs broadly is the limited funding available, often from various sources that are poorly coordinated with one another. To help organizations plan effectively to adopt EBPs, we conducted a review of recent evidence (i.e., since the passage of the 2010 Affordable Care Act) on strategies for financing EBP adoption in behavioral health systems. We present definitions of 23 identified strategies and describe each strategy’s reported (in the research literature) level of use to fund EBP adoption in behavioral health services. Of the 23 financing strategies, 13 strategies had evidence of use, 4 had potential for use, 5 had conceptual use only, and 1 was potentially contraindicated. Examples of strategies with evidence of use include increased fee-for-service reimbursement, grants, cost sharing, and pay-for-success contracts. This comprehensive list of EBP financing strategies may help guide decision-making by behavioral health professionals, system leaders, and policymakers. The article also presents a research agenda for building on the current research literature by (1) advancing methods to evaluate financing strategies’ effects, (2) partnering with stakeholders and decision-makers to examine promising financing strategies, (3) focusing on strategies and service systems with the greatest needs, (4) improving methods to guide the selection of financing strategies, and (5) paying greater attention to sustainable long-term financing of EBPs.

## The need for increased investment in evidence-based practices

Across the life span, as many as one in five people experience a mental health or substance use problem each year (Costello & Angold, 2016; Substance Abuse and Mental Health Services Administration, 2017), with billions of dollars in associated economic impact (Trautmann et al., 2016). Rigorous research has identified numerous evidence-based practices (EBPs) with demonstrated effectiveness for behavioral health problems (Society of Clinical Psychology, 2019; Weisz & Kazdin, 2017) that can also produce significant economic benefits when implemented on a large scale (Dopp et al., 2017, 2018; Okamura et al., 2018). Yet service systems continue to offer treatments of limited or unknown effectiveness (Bruns et al., 2016; Jones et al., 2014; McHugh & Barlow, 2010)—especially for marginalized and underserved groups (Agency for Healthcare Research and Quality, 2017). The U.S. behavioral health system needs reforms that make EBPs much more widely available than is currently the case, thus maximizing the population-level impact of services (Jones et al., 2014; Kazak et al., 2010).

A growing body of research identifies ways to improve the implementation of EBPs, where implementation means the initial adoption and spreading of EBPs in everyday clinical settings (Bauer et al., 2015). Many factors can influence implementation processes and outcomes, but limited and fragmented funding is often noted as a critical barrier to EBP implementation (Beidas et al., 2016; Lang & Connell, 2017; Raghavan et al., 2008). To help address that barrier, we conducted a scoping review to identify financing strategies that might support the implementation of EBPs in behavioral health services.

## Challenges involved in funding EBP implementation

Implementing EBPs is expensive for behavioral health provider agencies because it generally requires them to engage in many activities beyond typical service provision (Bruns et al., 2016; Chambers et al., 2013; McHugh & Barlow, 2010; Raghavan et al., 2008; Schoenwald et al., 2011), such as monitoring treatment outcomes, fidelity, and adaptations; delivery of non-routine services (e.g., case management, care coordination, and caregiver involvement); purchasing required resources and materials; and expert training and consultation. These activities are not only core to many EBPs but also result in numerous direct costs and indirect expenses (i.e., lost productivity or billable hours) that can be difficult for an agency to afford; subsequently, such core activities are often absent or low-quality in community services. Given the ongoing, dynamic influences of factors like clinician turnover (Beidas et al., 2016), continued implementation support is also necessary to sustain EBPs, representing a considerable ongoing investment (Bond et al., 2014; Roundfield & Lang, 2017).

Unfortunately, the funding needs of EBPs are not well aligned with typical methods of financing for behavioral health services, which rely heavily on siloed service delivery systems (e.g., mental health and substance use) seeking support from third-party funders (e.g., public and commercial insurers, government authorities, philanthropy; see Garfield, 2011). Funding for direct service delivery traditionally comes from program budgets and fee-for-service payments. Indeed, private and public payors currently account for nearly all U.S. health care expenditures (Cleverley & Cleverley, 2018; Folland et al., 2017), including behavioral health (Frank & Glied, 2006), and therefore funders have a tremendous influence on service delivery systems. Since these funds are often too limited to cover EBP delivery costs, let alone implementation (Knapp et al., 2006; Stewart et al., 2016), funders may disincentivize EBP implementation in favor of low-cost treatment options. This is especially true in behavioral health where patients often have little ability to pay for care out-of-pocket.

## Strategies to finance EBP implementation

Within the current funding context, maximizing the public health impact of EBPs will require implementation strategies that can align service delivery and funding systems to effectively support implementation activities (Knapp et al., 2006). Implementation strategies are methods or techniques used to enhance implementation, sustainment, or scale-up of an EBP (Proctor et al., 2013). Over the past decade, a national group of experts has begun a broader effort to compile and describe implementation strategies (Powell et al., 2015; Proctor et al., 2013; Waltz et al., 2015) to inform implementation research and practice.

That group of experts identified “financial strategies” as one important type of implementation strategy, but more work is needed to understand this subset of strategies. Close examination reveals that the “financial strategies” were often described very generally (e.g., “Fund and contract for the clinical innovation,” “Access new funding”; Powell et al., 2015) and grouped together simply because they involved money (Waltz et al., 2015). To clarify thinking in this area, we define financing strategies as those implementation strategies that secure and direct financial resources to support essential activities for EBP implementation; that is, strategies that link funding and service delivery systems. There remains a need for a list of financing strategies that are specified according to published guidelines (Proctor et al., 2013).

## The present study

The purpose of this scoping review was to assess the current state of scientific understanding for EBP financing strategies. Since neither behavioral health experts nor implementation experts have given much attention to this topic, a scoping review was the most appropriate literature review method to use. A scoping review assesses the current state of evidence for a topic area; such reviews are broad in nature and consider many sources of evidence (e.g., quantitative and/or qualitative research, policy documents), providing a general assessment as opposed to the more targeted questions posed in a systematic review (Tricco et al., 2018). Based on the results of our scoping review, we developed (1) a conceptual figure that describes our thinking about financing strategies and makes critical distinctions among them, other implementation strategies, and other financing activities; (2) a compilation of 23 financing strategies for implementation and associated evidence from available literature; and (3) a research agenda that calls on behavioral health and implementation experts to leverage and study these financing strategies with the goal of developing robust, sustainable financing for EBPs.

## Conceptualization of financing strategies

**Figure 1.**
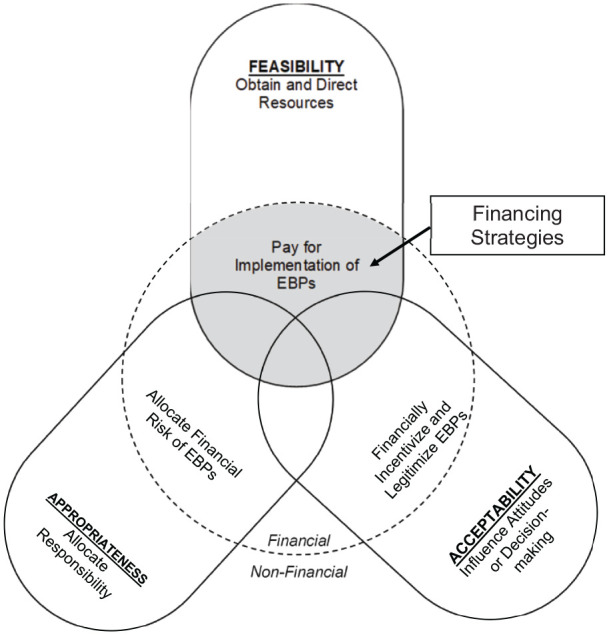
Conceptual figure of implementation financing strategies. *Note*. The three intersecting ovals represent key perceptual outcomes of implementation (acceptability, appropriateness, and feasibility) and the text included in each oval describes how implementation strategies target that outcome through financial (inside the dashed circle) and non-financial (outside the dashed circle) mechanisms. Financing strategies, indicated by gray shading, are any methods or techniques that seek to increase feasibility of evidence-based practices by obtaining and directing financial resources to support implementation.

Figure 1 depicts our conceptualization of financing strategies within the full range of EBP implementation strategies. We developed and refined this conceptual figure during our scoping review to capture our evolving conceptualization of financing strategies throughout the process. We present it here first to help orient readers to the concept of financing strategies.

We grounded our figure in three key outcomes that the implementation strategies influence: EBP acceptability, appropriateness, and feasibility (Proctor et al., 2011). Acceptability refers to perceptions of whether the EBP provides a reasonable option for delivering care—is it consistent with one’s personal and professional values? Appropriateness refers to perceptions of whether the EBP fits with the service delivery context and patient population—does it make sense to deliver it in our setting? Feasibility refers to perceptions of practical ability to adopt the EBP—can it be delivered in our setting, with current resources and constraints, to achieve desired clinical outcomes? These outcomes are perceptual (rather than behavioral) and thus, per expectancy theory (Georgopoulos et al., 1957), offer the most proximal indicators of whether a strategy can successfully promote EBP implementation by providers and decision-makers.

Figure 1 suggests that many implementation strategies can influence outcomes through non-financial mechanisms, such as influencing attitudes or decision-making (e.g., clinician education about the benefits of exposure therapy), helping to allocate responsibility (e.g., redefining roles in a clinic so that community health workers have the authority to deliver therapy), or obtaining and directing resources (e.g., hiring an EBP program coordinator or building additional office space). The area inside the dashed circle in Figure 1 represents the subset of strategies targeting each outcome that somehow involve monetary transactions (i.e., “financial strategies” in Waltz et al., 2015). We only define the subset of those strategies that involve obtaining and directing financial resources to pay for services—indicated by the gray shading—as financing strategies. Thus, we think of financing strategies as most closely aligned with increasing feasibility of implementation. Yet, the overlap among the three outcomes in the figure indicates our expectation that financing strategies could sometimes (but not always) influence appropriateness and acceptability as well.

An example that illustrates the multi-faceted nature of financing strategies is pay-for-performance (P4P)—a financing model that augments traditional fee-for-service systems by allowing for additional payments in response to achieving predetermined performance metrics (Garner et al., 2018). P4P can function as a financing strategy for implementation when the payments are structured to help cover the increased costs of implementing or continuing to provide the EBP. Examples include the payor, such as an insurance company, offering bonuses to clinics for each 6-month period in which they achieve a desired EBP implementation outcome—for example, a certain proportion of behavioral health providers meets fidelity requirements for cognitive-behavioral therapy for depression—or a clinical outcome—for example, a certain proportion of patients with major depressive disorder shows clinically significant improvement. Regardless of the targeted outcome, if the bonuses are paid to the clinics (rather than individual providers as in some P4P models), then the extra income could be used to cover the cost of implementing cognitive-behavioral therapy in that clinic. Covering costs in this manner certainly can increase feasibility. However, this P4P model might also impact acceptability if major funders start incorporating P4P incentives for cognitive-behavioral therapy into their reimbursement systems, which provides a social “legitimating” signal that adopting the EBP is desirable (Mendel & Scott, 2010). The requirements for P4P payments could also influence appropriateness, as a higher proportion shifts risk onto the clinics (i.e., greater likelihood of paying to implement the EBP but not receiving the bonuses) and off from the payor.

Under such conditions, P4P would fall into the very center of Figure 1 where acceptability, appropriateness, and feasibility converge within a single financing strategy. Of course, not every financing strategy is so comprehensive. Receiving a grant, contract, or philanthropic gift to implement an EBP would still qualify as a financing strategy but might not directly influence acceptability or appropriateness. Other financing strategies may only influence appropriateness or acceptability in addition to feasibility, but not both. Finally, it bears repeating that not all strategies that involve money are necessarily financing strategies. For example, financial incentives provided directly to the providers for EBP adoption and delivery can increase acceptability (Beidas et al., 2017), but we would only consider the incentives of a financing strategy if they helped to cover the organizational costs of the EBP. In fact, such a strategy has more in common with non-financial incentives (e.g., social praise) as both use contingencies to shape attitudes and decision-making regarding implementation.

## Financing strategies compilation

Our scoping review of EBP financing strategies covered two major sources of information. First, we reviewed existing compilations of implementation strategies or financing mechanisms to identify financing strategies and generated detailed descriptions of each financing strategy. Second, we conducted a literature search to identify implementation studies and related resources (e.g., reports and policy briefs) that involved financing strategies. The purpose of this step was to characterize the evidence available about each strategy’s use in behavioral health; we initially hoped to identify research evaluating the impact of financing strategies, but as described subsequently, we were only able to characterize reported levels of use. We documented our approach with the Preferred Reporting Items for Systematic Reviews and Meta-Analyses—scoping review checklist (Tricco et al., 2018; see Additional File 1).

### Review of existing compilations

Many of the details required to identify and characterize financing strategies are not typically reported in behavioral health implementation research (see Hooley et al., 2019, for a general discussion of implementation strategy reporting). Thus, we used our team’s collective knowledge of implementation science, behavioral health services, public administration/financing, health economics, and health policy to select compilations from which to identify financing strategies. Table 1 summarizes the nine compilations (three of implementation strategies and six of health care financing mechanisms) that we reviewed and the number of potential financing strategies identified in each compilation. When a given compilation was divided into sub-categories, we focused on the sub-categories most relevant to financing strategies; these are noted in the table. Overall, we considered 205 potential strategies, of which 118 (58%) were selected as candidates for inclusion in the financing strategies compilation. Strategies were selected if they met our aforementioned definition of a financing strategy: *methods or techniques that support implementation of EBPs by securing and directing financial resources*. For this stage of review, we considered all possible strategies without regard to their use in behavioral health. Strategies were excluded if they only involved payment for existing or routine care (without connection to EBP implementation); involved non-financial incentives or activities; or regulated purchasing (e.g., price setting).

**Table 1.** Results of financing strategy identification through review of existing compilations.

Compilation	Type	Reference(s)	Sub-categories reviewed	Number of strategies		
				Financing	Non-financing	Total
1. Cochrane EPOC: Effective Practice and Organization of Care	I	Cochrane EPOC (2002, 2015)	Financial interventions/ arrangements	19	18	37
2. ERIC: Expert Recommendations for Implementing Change	I	Powell et al. (2012, 2015); Waltz et al. (2015)	Financial strategies	23	0	23
3. HCP-LAN: Health Care Payment Learning and Action List	F	HCP-LAN (2017)	n/a	10	1	11
4. Mixed provider payment systems	F	Feldhaus and Mathauer (2018)	n/a	10	1	11
5. NASMHPD: National Association of State Mental Health Program Directors	F	NASMHPD (2010, 2012); Steverman & Shern (2013)	Financing mechanisms; changes in financing and payment policies	15	22	37
6. Policy Ecology Framework	I	Raghavan et al. (2008)	n/a	5	8	13
7. RAND Corporation health care financing resources	F	Friedberg et al. (2015); RAND Corporation (2015)	n/a	13	4	17
8. SAMHSA (Substance Abuse and Mental Health Services Administration) Medicaid handbook	F	SAMHSA (2013)	Reimbursement methodologies	7	19	26
9. Strategies to increase access to child health services	F	Bright et al. (2017)	n/a	16	14	30
All compilations				118	87	205

Type of compilation: F = health care financing, I = implementation strategies.

### Literature search techniques

The first author used three search procedures to identify potential articles for inclusion in our review. Articles were included if they described one or more financing strategies that were (1) used to pay for implementation of one or more behavioral health EBPs and (2) identified in our review of existing compilations. We did not require the articles had been published with peer review given that much information about policy-related interventions, such as financing strategies, is published outside of the peer-reviewed journal articles. As needed, inclusion decisions were discussed with the team of authors until consensus was reached.

First, we conducted keyword searches within various databases using a combination of keywords and search limits designed to capture the following concepts:

1. Financing strategies (Financ*, Fund*, Reimburse*, Pay*, Repay*, Renumerate*, Medicaid, Medicare);
2. Implementation (Implement*, Scale*, Spread, Deliver*, Uptake, Adopt*, Sustain*, Maintain*, Operat*);
3. Behavioral health (“Mental disorder”; “Mental health”; “Mental illness”; “Behavioral disorder”; “Behavioral health”; “Behavior problem*”; Psych*; “Disruptive behavior”; Trauma*).

The databases searched were PsycINFO, MEDLINE (via PubMed), and EconLit. It should be noted that our team initially focused our search strategies on youth behavioral health services, which is an area of interest for many of us, but we found that most search results focused on adult services. Therefore, we expanded our search strategies to include all age groups and eliminated search terms (e.g., “Family First” for the Family First Prevention and Services Act) and databases (e.g., National Children’s Alliance online library) that were not yielding unique results. We restricted all searches to the date range January 1, 2010 through March 31, 2019 (the date we completed the review) because research prior to the passage of the U.S. Patient Protection and Affordable Care Act in 2010 was likely to be incomplete or outdated.

Second, the first author identified promising articles by hand-searching tables of contents from 17 peer-reviewed journals, selected by the research team, that regularly publish studies about implementation and/or health care financing (e.g., *Administration and Policy in Mental Health and Mental Health Services Research, American Journal of Public Health, BMC Health Services Research, Health Affairs, Implementation Science, Journal of Behavioral Health Services Research*). For the sake of feasibility, this review was limited to the past 5 years. A list of health care financing articles from the RAND Corporation (www.rand.org/topics/health-care-financing.html) was also reviewed from 2010 to March 2019. Finally, the first author examined the reference lists from both potential articles and the previously reviewed compilations to identify additional articles.

**Figure 2.**
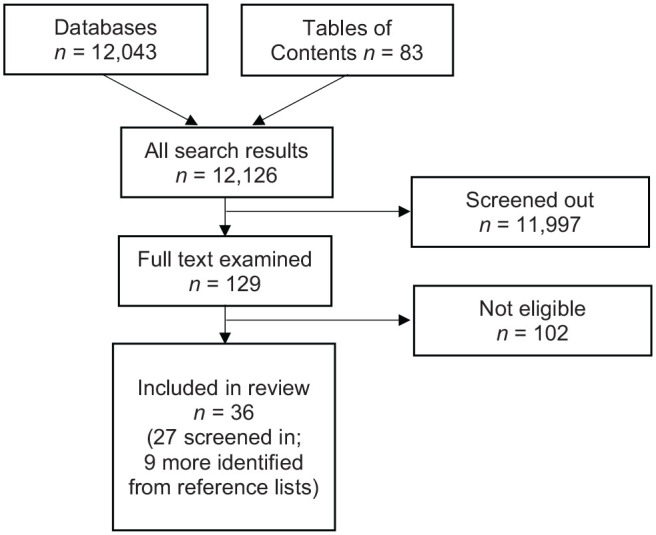
Sources of evidence screened for eligibility and included in the scoping review.

A flowchart for our literature search is included in Figure 2. Our search yielded 12,126 articles (12,043 from the database searches and 83 from the table of contents searches) to be reviewed for inclusion/exclusion. Most articles were screened out by a review of the title and abstract, with 129 receiving full-text review to evaluate eligibility criteria. Of those articles, 27 were selected for inclusion and review of their reference lists generated an additional nine articles that were also included. The final list of 36 articles (indicated in the reference list) served as the evidence base for financing strategies used in behavioral health.

### Creating the financing strategies compilation

Once the literature search was complete, we reviewed all materials (i.e., strategy definitions from the compilations; articles identified in the literature search) to create a comprehensive compilation of financing strategies used in behavioral health, including detailed descriptions and the level of use for each strategy. The first author created the original compilation, after which the co-authors reviewed and provided formative feedback. The study team iteratively revised the compilation until consensus was reached.

#### Descriptions of financing strategies

To create the compilation, we selected a final list of financing strategies and generated detailed descriptions of each strategy along key dimensions. The 118 strategies identified from our nine source compilations each had their own definitions, and there was often considerable overlap—but also key distinctions—among the strategies and definitions identified, so we combined overlapping strategies. We then identified the characteristics of each financing strategy using seven key dimensions for implementation strategies (i.e., actors, actions, action targets, temporality, dose, outcomes addressed, and justification) as outlined by Proctor et al. (2013) and then synthesized those characteristics into an overall definition. We reviewed the 36 articles identified in our literature review as part of our process for creating the compilation of financing strategies; it was useful to consider how articles had applied and described different strategies to assist in identifying their key dimensions.

Ultimately, we identified 23 financing strategies. Table 2 lists the strategies, their definitions, and the references from which they were identified. We aimed to create definitions that clearly distinguished among strategies (e.g., highlighting key differences between blended [combined] and braided [coordinated] funding streams) but were also general enough that they could be applied across a variety of contexts and situations (e.g., the definitions for both blended and braided funding streams could describe organization of funds from a variety of government agencies). As a complement to that table, Table 3 presents a detailed specification of the seven key dimensions (Proctor et al., 2013) that we identified for each financing strategy.

**Table 2.** Financing strategy compilation: definitions and primary sources.

Financing strategy	Definition^a^	Primary source(s)^b^	Reported level of use^c^
Fee-for-service reimbursement	Include the EBP in an insurance fee-for-service list/formulary so that providers can receive reimbursement for providing that practice	1, 2, 3, 4, 5, 6, 7, 8	Current use
Increased fee-for-service reimbursement rate	Increase insurance reimbursement rates to providers for the EBP, relative to other services, to offset increased costs to providers for delivering that practice	2, 3, 6	Current use
Removed/altered billing limits	Allow insurance payments to providers for services that are disallowed under typical billing limits (additional sessions, services for patients with designated diagnoses, etc.) when delivering the EBP	2, 5, 7, 8	Current use
Technical support for billing	Provide assistance (from insurance or a third party) to providers for preparing and successfully submitting claims for the delivery of the EBP	2	Current use
Pay-for-success (PFS) financing	Establish agreements in which private or non-profit investors prospectively provide funding for providers to deliver the EBP, and a government entity provides a payout to the investors if pre-established outcomes or quality metrics are achieved in a designated time period	5	Current use
Global budgets for general funds	When allocating government general funds, include funding in the overall annual budget of a state, county, or municipal agency to cover the costs of the agency’s providers to deliver the EBP	1, 5	Current use
Line-item budgets for general funds	When allocating government general funds, include designated (protected) funding in the annual budget of a state, county, or municipal agency to cover the costs of the agency’s providers to deliver an EBP	1, 5	Current use
Braided funding streams	Coordinate multiple funding sources across two or more government agencies to cover the costs of the agencies’ providers to deliver the EBP, such that each individual funding source remains accounted for separately; generally established through annual budgets	5	Current use
Contracts for EBPs	Award funding contracts from state, county, or municipal agencies to provider organizations that agree to deliver the EBP	1, 2, 5, 6, 9	Current use
Inclusion in block grants	Allow payment to providers for delivering the EBP using block grants, which provide a fixed amount of money to state, county, or municipal agencies to pay for a designated set of health services	2, 5	Current use
Shifting funds between programs	Reallocate funds from other programs and practices, within or across state, county, or municipal agencies, to cover the costs of delivering the EBP; often funds currently dedicated to services whose use is expected to decline due to the EBP	2	Current use
Grant funding	Award funding from government or private grant-making agencies to provider organizations that propose to deliver the EBP	1, 2	Current use
Charitable/philanthropic donations	Collect donations from private or non-profit investors that will be used by provider organizations to cover the costs of delivering the EBP	1, 2, 5	Current use
Cost sharing	Use state, county, or municipal funds to pay for infrastructure (such as a dedicated intermediary organization) that supports the delivery of EBPs by providing training, technical assistance, and other non-monetary resources to providers within a given jurisdiction	2	Current use
Credentialing/rostering providers	Establish agreements between insurance companies and training organizations for EBPs, in which designated providers are allowed to receive payment for the EBP (often at an increased rate)	6	Potential use
Blended funding streams	Combine multiple funding sources across two or more government agencies to cover the costs of the agencies’ providers to deliver the EBP, such that all funding is accounted for together and no longer separable by source; generally established through legislative action	5	Potential use
Dedicated taxes	Collect a state, county, or municipal tax, then allocate the revenue from that tax to provider organizations that deliver the EBP	1, 2, 5	Potential use
Capitated or patient-based payments	Provide a set, prospective insurance payment to providers that is expected to cover all expenses for a given patient’s care (including increased costs of delivering the EBP) in a designated time period	1, 2, 3, 4, 7, 8	Conceptual only
Pay-for-performance (P4P)	Provide a financial bonus (on top of other insurance payments) to provider organizations for achieving pre-established outcomes or quality metrics in a designated time period, with bonuses sufficient to cover increased costs to providers for delivering the EBP	1, 2, 3, 4, 7, 9	Conceptual only
Value-based purchasing	Provide insurance reimbursement to providers only when they achieve pre-established outcomes or quality metrics for a designated time period, with reimbursement sufficient to cover increased costs to providers for delivering the EBP	3, 7	Conceptual only
Government bonds	Issue state, county, or municipal bonds (securities) for purchase by private or non-profit investors, then allocate the revenue from those bonds to provider organizations that deliver the EBP	5	Conceptual only
Vouchers	Provide vouchers of a predetermined value that patients can redeem to receive the EBP from providers, who are then repaid by the insurance or government payor that issued the voucher	1	Conceptual only
Bundled or episode-based payments	Provide a set insurance payment to a group of providers that is expected to cover all expenses for a given diagnosis or episode of care (including increased costs of delivering and coordinating the EBPs across providers); can be prospective or retrospective payments	1, 3, 4, 5, 7, 8	Potentially contraindicated use

*Note.* EBP = evidence-based practice.

a Definitions were derived from seven key dimensions of implementation strategies, which are presented for each financing strategy in Table 3.

b The numbers for primary sources refer to the list of existing compilations from which we identified financing strategies (see Table 1 for more details).

c Refers to the reported level of use for each financing strategy in behavioral health services, within the research literature identified in our scoping review (see Table 4 for more details).

**Table 3.** Financing strategy compilation: detailed characteristics of strategies.

Financing strategy	Key characteristics^a^						
	Actors	Actions	Action targets	Temporality	Dose	Outcomes	Justification
Fee-for-service reimbursement	Insurance or managed care companies	Provide reimbursement for a specified EBP (i.e., include on list/formulary)	Provider organizations	Retrospective (reimbursement)	Per billable unit of the EBP	Decrease costs for service providers; increase acceptability of EBP	Reimbursement is necessary to cover the costs of care; excluding EBPs from fee-for-service lists/formularies disincentivizes their use
Increased fee-for-service reimbursement rate	Insurance or managed care companies	Provide increased reimbursement for a specified EBP, relative to other services	Provider organizations	Retrospective (reimbursement)	Per billable unit of the EBP	Increase feasibility and decrease cost for service providers	EBPs have considerable out-of-session costs—such as for training, consultation, case management, and so on—for which traditional fee-for-service reimbursement is insufficient, so increased reimbursement rates are needed to make EBPs viable in a fee-for-service model
Removed/altered billing limits	Insurance or managed care companies	Provide payment for a specified EBP in cases where payment would not otherwise be provided (e.g., agree to a waiver)	Provider organizations	Variable	Variable	Increase feasibility and decrease cost for service providers	In some cases, the value of an EBP can only be achieved by allowing for payment in greater frequency or with different patients than is typically allowed by the funding agreement, so altering the billing limits is necessary to make the EBP feasible
Technical support for billing	Insurance or managed care companies, or a third party	Provide assistance that increases the likelihood of successful billing for an EBP	Providers, provider organizations	Variable	Variable	Increase feasibility and decrease cost for service providers	Providers may not be able to successfully submit claims for an EBP without technical assistance, and they cannot obtain the benefits of funding agreements without successful billing
Pay-for-success (PFS) financing	Private or non-profit investors, government payor, independent evaluation team, third-party intermediary (optional)	Private investors fund implementation of an EBP; government payor provides payout to investors if pre-established outcomes/quality metrics achieved (per independent evaluation) in a designated time period; may be managed by an intermediary	Provider organizations (receive funding), investors (receive payout)	Prospective (funding), retrospective (payout)	Funding 1×, but payout structure is variable	Increase adoption and penetration of EBP; increase feasibility and decrease cost for service providers and government payor	Provides the initial investment for implementation of an EBP (e.g., covering costs of training, consultation, materials, space, system reorganization), which is often beyond the resources available to many government agencies, and ensures that the government pays for interventions that produce measurable value to the public (shifts financial risk to investors)
Global budgets for general funds	State, county, or municipal agencies	Include funding to cover the costs of EBP as part of responsible agency’s overall budget when allocating general funds	Provider organizations	Prospective	Per year	Decrease costs for service providers; increase acceptability of EBP	Budget allocations are necessary to cover the costs of care, including service provision and out-of-session costs (e.g., training, consultation, case management); failure to budget for EBPs disincentivizes their use
Line-item budgets for general funds	State, county, or municipal agencies	Include designated (protected) funding for EBP in responsible agency’s budget when allocating general funds	Provider organizations	Prospective	Per year	Decrease costs for service providers; increase acceptability of EBP	Budget allocations are necessary to cover the costs of care, including service provision and out-of-session costs (e.g., training, consultation, case management); failure to budget for EBPs disincentivizes their use and line-item budgets protect the funds for use with a designated EBP
Braided funding streams	(At least two) state, county, or municipal agencies	Coordinate multiple funding sources to pay for an EBP, such that each individual funding source remains accounted for separately; generally established through annual budgets	Provider organizations	Prospective	Per year	Increase feasibility and decrease cost for service providers and participating agencies	EBPs accrue costs and benefits across multiple government entities; braided funding streams allow for the distribution of costs across entities to align with the distribution of benefits, with higher up-front feasibility and lower long-term feasibility
Contracts for EBPs	State, county, or municipal agencies	Award contracts to organizations that agree to provide an EBP	Provider organizations	Variable	Variable number of years; payment may be fee-for-service	Increase feasibility and decrease costs for service providers; increase acceptability of EBP	Allocates funds to cover the costs of EBPs, including service provision and out-of-session costs (e.g., training, consultation, case management); making EBP a requirement for contract funding incentivizes their use
Inclusion in block grants	State, county, or municipal agencies	Provide payment for an EBP using grants that provide a fixed amount of money to pay for a designated set of health services	Provider organizations	Variable	Variable; payment may be fee-for-service	Decrease costs for service providers; increase acceptability of EBP	Funding is necessary to cover the costs of care; allowing payment for EBP from block grants incentivizes their use
Shifting funds between programs	State, county, or municipal agencies	Pay for an EBP using funds that were previously allocated to a different program or practice	Provider organizations	Prospective	Variable	Decrease costs for service providers; increase acceptability of EBP	Reallocation of funds can cover the costs of care, including service provision and out-of-session costs (e.g., training, consultation, case management); funds are often reallocated from programs and practices whose use is expected to decline due to the EBP
Grant funding	Government or private grant-making agencies	Award grant funds to organizations that propose to provide an EBP	Provider organizations	Variable	Variable number of years	Increase feasibility and decrease costs for service providers; increase acceptability of EBP	Provides funds to cover the costs of EBPs, including service provision and out-of-session costs (e.g., training, consultation, case management); making an EBP a requirement for grant funding incentivizes its use
Charitable/ philanthropic donations	Private or non-profit investors	Donate funds to organizations that will provide an EBP	Provider organization	Variable	Variable	Increase feasibility and decrease costs for service providers; increase acceptability of EBP	Provides funds to cover the costs of EBPs, including service provision and out-of-session costs (e.g., training, consultation, case management); when donors make EBP a condition of the gift, that incentivizes the use of those practices
Cost sharing	State, county, or municipal agencies	Pay for infrastructure to support the delivery of EBPs through training, technical assistance, and other non-monetary resources	Provider organization; intermediary organizations and resources	Prospective	Variable	Increase feasibility and decrease costs for service providers	Provides resources necessary to support the provision of EBP (e.g., training, consultation) that may be difficult for provider agencies to purchase or may be purchased at a better economy of scale by the government
Credentialing/ rostering providers	Purveyor or intermediary organizations, insurance, or managed care companies	Designate providers as eligible to receive increased reimbursement for an EBP	Providers, provider organizations	Retrospective (reimbursement)	1× or more frequently (e.g., annually) if recertification required	Increase fidelity to EBP; increase feasibility and decrease cost for service providers	Payors only provide reimbursement for the EBP to approved providers, who are expected to provide the practice with greater fidelity and thus are worth the increased cost of services
Blended funding streams	(At least two) state, county, or municipal agencies	Combine multiple funding sources to pay for an EBP, such that all funding is consolidated and no longer separable by source; generally established with legislative action	Provider organizations	Prospective	Variable, depends on enacting legislation	Increase feasibility and decrease cost for service providers and participating agencies	EBP accrue costs and benefits across multiple government entities; braided funding streams allow for the distribution of costs across entities to align with the distribution of benefits, with lower up-front feasibility and higher long-term feasibility
Dedicated taxes	State, county, or municipal government	Collect taxes that are allocated to agencies and providers to deliver the EBP	Provider organization	Prospective	Per year	Increase feasibility and decrease costs for service providers	Taxes provide additional revenue that can be used to cover the costs of EBPs (e.g., training, consultation, case management), thus returning value to taxpayers
Capitated or patient-based payments	Insurance or managed care companies	Provide a set payment that is expected to cover all expenses for a given patient’s care in a designated time period	Provider organizations	Prospective (pre-payment)	Per month or per year	Increase appropriateness of care; *can* increase feasibility and decrease cost for service providers, depending on structure	Provides funds necessary to ensure high-value care for a given patient, while shifting financial risk management to providers; if the capitated rate is sufficiently high, it could be used to cover considerable out-of-session costs of EBPs—such as for training, consultation, case management, and so on—especially because it is provided prospectively
Pay-for-performance (P4P)	Insurance or managed care companies	Provide a financial bonus (on top of other payments) for achieving pre-established outcomes or quality metrics in a designated time period	Providers, provider organizations	Retrospective	Per month or per year	Increase acceptability of the EBP; *can* increase feasibility and decrease cost for service providers, depending on the structure	Provides incentives for providing high-quality care, which can be tied directly or indirectly to an EBP; incentives to the provider organization could be used to cover considerable out-of-session costs of EBPs—such as for training, consultation, case management, and so on
Value-based purchasing	Insurance or managed care companies	Provide reimbursement only when pre-established outcomes or quality metrics are achieved	Providers, provider organizations	Retrospective	Per month or per year	Increase appropriateness of care; *can* increase feasibility and decrease cost for service providers, depending on the structure	Requires evidence of high-value care prior to reimbursement, shifting financial risk management to providers; if the reimbursement rate is sufficiently high, it could be used to cover considerable out-of-session costs of EBPs—such as for training, consultation, case management, and so on
Government bonds	State, county, or municipal agencies; private or non-profit investors	Government entity sells bonds (securities) to investors, and the income is allocated to provide an EBP	Provider organization	Prospective	Per year	Increase feasibility and decrease costs for service providers	Bonds provide additional revenue that can be used to cover the costs of EBPs (e.g., training, consultation, case management), thus returning value to taxpayers
Vouchers	Insurance or managed care companies; state, county, or municipal agencies	Provide vouchers of a predetermined value that patients can redeem for EBP from providers	Patients, provider organizations	Retrospective	Per billable unit of the EBP	Decrease costs for service providers; increase acceptability of EBP	Vouchers can cover the costs of care, including service provision and out-of-session costs (e.g., training, consultation, case management); restriction of voucher use to EBPs can incentivize the use of such practices
Bundled or episode-based payments	Insurance or managed care companies	Provide a set payment that is expected to cover all expenses associated with a given diagnosis or episode of care (across providers and provider organizations)	Provider organizations	Prospective or retrospective	Per care episode	Increase appropriateness of care; *can* increase feasibility and decrease cost for service providers, depending on the structure	Provides funds necessary to ensure high-value care for a given clinical problem, while shifting financial risk management to providers; if the bundled rate is sufficiently high, it could be used to cover considerable out-of-session costs of EBPs—such as for training, consultation, case management, and so on—especially when it is provided prospectively

*Note.* EBP = evidence-based practice. ^a^As defined by Proctor et al. (2013).

#### Reported levels of use for financing strategies in the research literature

Through our review of the 36 articles identified in the literature search, we characterized the use of each financing strategy—as documented in existing research—to fund implementation of evidence-based behavioral health services. Given the state of the literature, it was not possible to provide a detailed assessment of the impact of financing strategies, as might be done in a systematic review. All the identified studies used observational methods that could not evaluate the impact of discrete financing strategies. Instead, for each article, we recorded the (1) financing strategy(ies) described and (2) service system(s) involved, both behavioral health (mental health, substance use, intellectual/developmental disability) and others (criminal/juvenile justice, child welfare, health care/medical, dental, public health education, social services [e.g., employment, housing]). We used that information to categorize each strategy into one of the four general levels:

1. **Current use:** Evidence from three or more articles that the strategy has been used to fund the implementation of evidence-based behavioral health services.
2. **Potential use:** Evidence that the strategy has been used to fund the implementation of evidence-based health services in general, but limited or absent evidence (two or fewer articles) for its use in behavioral health services.
3. **Conceptual only:** Strategy was identified in our review of existing compilations and thus could be promising to explore in future research, but we found no articles describing its use to fund the implementation of any evidence-based health services.
4. **Potentially contraindicated use:** Some research indicates the strategy may not be feasible or effective for funding the implementation of evidence-based behavioral health services, at least under certain conditions (no strategies were clearly contraindicated).

Of the 36 articles, 14 (38%) mentioned multiple financing strategies for a mean of 2.4 financing strategies per article (range = 1–8; *SD* = 2.2). Furthermore, 28 articles (78%) discussed the use of financing strategies in behavioral health systems. Each article examined between one and six service systems, with 15 (42%) considering multiple systems and an average of 1.8 systems per article (*SD* = 1.4).

Table 4 summarizes the level of use for each financing strategy as reported in the research literature. The table presents all information from our review and specifically highlights the evidence of use within behavioral health systems. Overall, 13 financing strategies (56%) had evidence of use within behavioral health services, 4 (17%) had potential for use, 5 (23%) were only conceptual, and 1 (4%) was potentially contraindicated. Among strategies in the two highest levels of use, all 17 had evidence of use in mental health services, 9 (53%) in substance use services, and 1 (6%) in intellectual/developmental disability services. Criminal/juvenile justice (*n* = 9; 53%) and child welfare (*n* = 13; 76%) were the most common non-behavioral-health service systems in which financing strategies were used.

**Table 4.** Financing strategy compilation: summaries of levels of use in behavioral health services.

Reported level of use^a^	Financing strategy^b^	Service systems using strategy^c^		Reference(s)^d^
		Behavioral health	Other	
Current use	Fee-for-service reimbursement	MH, SA	CJ, CW, DE	*Gottfredson et al. (2018); Herndon et al. (2015); *Powell et al. (2016); *Rieckmann et al. (2015); *Scudder et al. (2017); *Stroul et al. (2007, 2009)
	Increased fee-for-service reimbursement rate	MH	CJ, CW	*Bruns et al. (2016); *Covell et al. (2016); *Dopp et al. (2018); *Jones et al. (2014); *Magnabusco (2006); *Powell et al. (2016); *Scudder et al. (2017); *Stroul et al. (2007, 2009)
	Removed/altered billing limits	ID, MH	SS	*Miller et al. (2016); *Powell et al. (2016); Szanton et al. (2015); *Velott et al. (2016)
	Technical support for billing	MH	CW	*Magnabusco (2006); *Powell et al. (2016); *Scudder et al. (2017)
	Pay-for-success (PFS) financing	MH, SA	CJ, ED, HC, PH, SS	*Fraser et al. (2018); *Iovan et al. (2018); Katz et al. (2018); *Lantz et al. (2016); *Tan et al. (2015)
	Global budgets for general funds	MH, SA	CJ, CW, ED, HC, SS	*Armstrong (2012); *Jaramillo et al. (2018); *Jones et al. (2014); Nagle & Usry (2016); *Rieckmann et al. (2015); *Stroul et al. (2007, 2009)
	Line-item budgets for general funds	MH, SA	CJ, CW, ED, HC, SS	*Armstrong (2012); *Jaramillo et al. (2018); *Jones et al. (2014); Nagle & Usry (2016); *Rieckmann et al. (2015); *Stroul et al. (2007, 2009)
	Braided funding streams	MH, SA	CW	AGA (2014); *Jaramillo et al. (2018); Nagle & Usry (2016); *Stroul et al. (2007, 2009)
	Contracts for evidence-based practices (EBPs)	MH, SA	CW	*Bruns et al. (2016); *Jaramillo et al. (2018); *Magnabusco (2006); *McBeath et al. (2018); *Powell et al. (2016); *Rieckmann et al. (2015); *Scudder et al. (2017); *Stroul et al. (2007, 2009); *Willging et al. (2016)
	Inclusion in block grants	MH, SA	CJ, CW, SS	*Armstrong (2012); *Jaramillo et al. (2018); *Magnabosco (2006); *Rieckmann et al. (2015); *Stroul et al. (2007, 2009)
	Shifting funds between programs	MH	CJ, SS	*Armstrong (2012); *Magnabusco (2006); *Stroul et al. (2007, 2009)
	Grant funding	MH, SA	CJ, CW, PH, SS	*Apsler et al. (2017); *Armstrong (2012); *Edwards et al. (2015); *Magnabosco (2006); *Orwin et al. (2014); *Rieckmann et al. (2015); *Scudder et al. (2017); *Stroul et al. (2007, 2009)
	Charitable or philanthropic donations	MH	CW	*Jaramillo et al. (2018); *Jones et al. (2014); *Magnabusco (2006); *Stroul et al. (2009)
	Cost sharing	MH	CJ, CW	*Bruns et al. (2016); *D’Angelo et al. (2017); *Jones et al. (2014); *Magnabusco (2006); *NCTSN (2016); *Powell et al. (2016); *Stroul et al. (2007, 2009)
Potential use	Credentialing/rostering providers	MH	–	*NCTSN (2016)
	Blended funding streams	MH	CW	AGA (2014); *Jaramillo et al. (2018); Nagle & Usry (2016)
	Dedicated taxes	MH	CW	*Jaramillo et al. (2018); *Stroul et al. (2009)
Conceptual only	Capitated or patient-based payments	–	–	
	Pay-for-performance (P4P)	SA	HC	Damberg et al. (2014); *Garner et al. (2012, 2018); Nagle & Usry (2016)
	Value-based purchasing	–	–	
	Government bonds	–	–	
	Vouchers	–	–	
Potentially contraindicated use	Bundled or episode-based payments	–	DE, HC	Damberg et al. (2014); Hussey et al. (2011); Niederman et al. (2017)

a Refers to the reported level of use of the financing strategy in behavioral health services, within the research literature identified in our scoping review. *Current use*: Evidence from three or more articles that the strategy has been used to fund the implementation of evidence-based behavioral health services. *Potential use*: Evidence that the strategy has been used to fund the implementation of evidence-based health services in general, but limited or absent evidence (two or fewer articles) for its use in behavioral health services. *Conceptual only*: Strategy was identified in our review of existing compilations, but we found no research articles describing its use to fund implementation of any evidence-based health services. *Potentially contraindicated use*: Some research indicates the strategy may not be feasible or effective for funding implementation of evidence-based behavioral health services, at least under certain conditions (no strategies were clearly contraindicated).

b Definitions for each financing strategy are presented in Table 2.

c Refers to the service system(s) documented in empirical literature—listed in the References column—as using the financing strategy. For behavioral health systems, ID = intellectual/developmental disability; MH = mental health; SU = substance use. For other systems, CJ = criminal/juvenile justice; CW = child welfare; DE = dental; ED = education; HC = health care/medicine; PH = public health; SS = social services (e.g., housing, employment).

d References marked with an asterisk (*) reported the use of the financing strategy in one or more behavioral health systems. AGA = Association of Government Accountants; NCTSN = National Child Traumatic Stress Network.

## Discussion

A major challenge to widespread, sustainable implementation of EBPs concerns how to cover the costs of implementation with the limited and fragmented funding available (Beidas et al., 2016; Lang & Connell, 2017; Raghavan et al., 2008). In this scoping review, we considered how financing strategies might help overcome the cost-related barriers to the implementation of EBPs that can, in turn, alleviate the public health and societal impacts of behavioral health problems. We identified a critical gap in the literature: researchers need to conduct more studies that advance the understanding of EBP financing strategies. This result was not our hope at the outset of the review, as we wished to identify more definitive evidence-based recommendations for financing implementation. Nevertheless, our conceptual figure and compilation of financing strategies represent important advances for implementation research in behavioral health that can directly inform future research, in ways described later in our proposed research agenda.

### Implications of the scoping review findings

We developed a conceptual figure (see Figure 1) that defines financing strategies as techniques that secure and direct financial resources to support EBP implementation. Previous work did not clearly distinguish financing strategies from related approaches to implementation or health care financing (see Table 1), resulting in a lack of clarity about approaches to financing EBPs. We encourage experts in implementation, behavioral health, and health care finance to use our conceptual figure to ensure adequate consideration of financing for implementation-related activities, such as covering EBP costs that are not included in traditional reimbursement or paying for implementation strategies. Given the highly fragmented nature of the current U.S. health care funding system (Cleverley & Cleverley, 2018; Folland et al., 2017; Garfield, 2011) and ongoing reliance on fee-for-service reimbursement models in behavioral health systems, implementation-related costs are rarely considered explicitly and are often treated as “someone else’s problem.” More explicit attention to financing strategies—which includes understanding their role within health care finance—could help service delivery and funding systems achieve greater alignment around funding for implementation activities (Knapp et al., 2006).

We identified 23 financing strategies for implementing EBPs in our review, of which 17 were reported having been used to at least some degree in behavioral health systems. No previous compilations included all 23 of these strategies; Powell et al. (2012, 2015) included the most, 13 strategies or 56.5% (see Table 2). Thus, this compilation appears to be the most comprehensive list of financing strategies for behavioral health care to date. We also used reporting guidelines for implementation strategies (Proctor et al., 2013) to provide comprehensive, standardized definitions of every financing strategy—a practice that remains rare in behavioral health research (Hooley et al., 2019)—and we identified the types of behavioral health services in which each strategy has been studied (see Tables 2 to 4). We encourage implementation researchers to use our compilation to define, measure, and report on the financing strategies used in their studies whenever feasible.

Our compilation may also serve as a useful resource for behavioral health professionals, system leaders, and policymakers who are interested in supporting the implementation of EBPs. The compilation offers a menu of options that these decision-makers might consider as part of strategic planning processes (Schell et al., 2013) that determine when and how to allocate resources toward implementation. For example, the compilation can be used by (1) the director of a behavioral health agency to identify alternative sources of financing to cover non-reimbursable EBP costs; (2) an intermediary agency to advise organizations whom they are supporting in the implementation process; (3) a state behavioral health director when deciding how to distribute resources to agencies within the state to encourage EBP adoption; or (4) an insurance company seeking to make changes in their reimbursement model to reward the quality and effectiveness of services provided. Of course, we recognize the need to further develop our compilation so that it offers more user-friendly information to decision-makers, such as guidance on the pros and cons of these strategies (e.g., importance vs. feasibility) and on how to execute them within their service systems.

### Proposed agenda for future research on financing strategies

Our financing strategy compilation and accompanying conceptual figure may prove necessary and useful, but we are certain that they are not sufficient. Our review provides numerous examples of how, in the present environment, EBP implementation costs remain daunting for even the most forward-thinking behavioral health systems to finance (see e.g., the case studies presented by Stroul, 2007, 2009). Additional efforts will be needed to fully align funding in ways that support EBP implementation. Therefore, we suggest a research agenda that will help to better understand financing strategies in terms of five key questions raised by the findings of our review: (1) How can we evaluate the effectiveness of financing strategies?; (2) Besides research literature, what are the other critical sources of evidence about financing strategies?; (3) Which strategies and systems have the greatest needs for future research in this area?; (4) Can financing strategies be combined and coordinated to increase their impact?; and (5) How can financing strategies support the long-term sustainment of EBPs?

First, we need to advance the methods used to evaluate financing strategies, as the research literature we reviewed was silent on this topic. We were able to describe whether the strategies had been used in behavioral health research studies, but found no studies that evaluated their effects on key implementation outcomes, such as adoption, feasibility, penetration, or sustainment (Proctor et al., 2011)—let alone health outcomes. Given that financing strategies are generally system-level interventions that involve changes in local, state, or even federal policy, it will be important for researchers to use policy research methods that can evaluate causality in quasi-experimental designs (e.g., non-equivalent dependent variables, which capture the effects of confounders; Coryn & Hobson, 2011) and represent complex relations within systems (e.g., qualitative and mixed methods [Eisman et al., 2020]; systems science methods, such as system dynamics or agent-based modeling [Luke et al., 2018]). Traditional research methods for evaluating the impact of individually focused interventions and implementation strategies (e.g., training) will rarely be sufficient for understanding the effects of financing strategies. However, it will still be important to ground models and hypotheses for evaluating financing strategies within the theories and frameworks from relevant disciplines (e.g., public finance, health economics, and implementation science).

Second, because financing strategies were not well-represented in the research literature we reviewed, we recommend considering alternative sources of evidence used by decision-makers to determine which financing strategies to offer and pursue under various circumstances. Such decision-makers could include behavioral health professionals, system leaders, and policymakers. Innovative new strategies may emerge in response to rapidly changing financial and regulatory environments, and decision-makers may be more influenced by pragmatic local evidence than they are by the results of published research studies. In future work, we plan to have representatives from behavioral health provider agencies, EBP intermediary agencies, and funding agencies to review our financing strategy compilation and, using the modified Delphi method developed for the Expert Recommendations for Implementing Change project (Powell et al., 2015; Waltz et al., 2014), provide feedback on (1) the current strategies and definitions and (2) additional strategies or sources of evidence to consider. It could also be worthwhile to conduct scoping reviews of financing strategy literature from other evidence sources that are more difficult to systematically search, such as health care trade publications or behavioral health system evaluation reports.

Third, although there is great need for more research on financing strategies in all behavioral health care, our review showed that the need is especially pronounced for certain strategies and service systems. Both substance use and intellectual/developmental disability services had less evidence of financing strategy use than did child welfare (i.e., a non-behavioral-health service system). It will be important to determine whether alternative search strategies (e.g., different search terms or databases) could produce more evidence of financing strategies for these systems. Also, because all the research we reviewed came from the United States, studies are needed to examine the use and impact of various financing strategies in other countries with different funding systems for health care. Moreover, the financing strategies classified as “conceptual only” and “potentially contraindicated” merit additional investigation. For example, we noted in the introduction that P4P holds promise as an EBP financing strategy—yet we found no evidence that such a P4P contract has been executed, let alone rigorously evaluated (Damberg et al., 2014; Garner et al., 2012, 2018; Nagle & Usry, 2016). Bundled payments also deserve more attention; we classified that strategy as “potentially contraindicated” because it has proven difficult to execute (Hussey et al., 2011), but ongoing advances could make bundled payments more feasible (Damberg et al., 2014; Niederman et al., 2017) and they are desired by behavioral health systems (Stroul, 2007; Stroul et al., 2009).

Fourth, we foresee an increasing demand for guidance on the tailored selection of financing strategies, which involves matching strategies to the goals, strengths, and needs of a given EBP implementation effort. The articles from our literature review described using as many as eight financing strategies (*M* > 2) in a given implementation effort (see e.g., Armstrong et al., 2012; Jaramillo et al., 2018; Powell et al., 2016; Rieckmann et al., 2015; Scudder et al., 2017) and indicated that stakeholders found coordination among various funding sources to be a major barrier to using EBPs in their systems (e.g., Jaramillo et al., 2018; Stroul, 2007; Stroul et al., 2009). Barring major reform of financing practices, behavioral health systems will require support to successfully incorporate the optimal combination of financing strategies for the EBPs they implement. Methods of tailoring implementation strategies are in their infancy, but a recent paper (Powell et al., 2017) identified several promising approaches for consideration. For example, we have started to explore how intervention mapping (Bartholomew et al., 1998)—a multi-step method for developing interventions or implementation strategies based on theory, research evidence, and stakeholder perspectives—can be used to guide stakeholders in their strategic selection of financing strategies for EBP implementation.

Finally, nearly all the research we reviewed on financing strategies has focused on funding for active EBP implementation. Such funding, although limited and potentially declining (Bruns et al., 2016), is regularly available at present through short-term government or foundation awards aimed at service “transformation” (Garfield, 2011; Scudder et al., 2017; Sigel et al., 2013). Yet recent research has revealed a need for much greater emphasis on the sustainment of EBPs (Scheirer & Dearing, 2011; Schell et al., 2013; Shelton et al., 2018), where sustainment is defined as continued use of an EBP over a specified period for the continued achievement of program and population outcomes (Scheirer & Dearing, 2011). Without sustained use after implementation, the public health impact of EBPs will remain limited. At present, financial barriers have led to unsuccessful sustainment in many EBP initiatives (Massatti et al., 2008; Rodriguez et al., 2018; Stewart et al., 2016) as the need for continued funding of implementation-related activities is substantial and difficult to satisfy (Bond et al., 2014; Roundfield & Lang, 2017). A few initial studies have laid the groundwork for understanding how financing strategies might influence sustainment (Apsler et al., 2017; Edwards et al., 2015; Scudder et al., 2017) but have also made it clear that more research—and different strategies—are needed to navigate the complex, multi-level, and ever-shifting funding environment as part of EBP sustainment efforts (Chambers et al., 2013; Shelton et al., 2018; Willging et al., 2015). We encourage greater attention in ongoing efforts around the goal of supporting sustainability planning for EBPs.

## Conclusion

In sum, the existing literature on EBP financing strategies in behavioral health offers promising solutions but raises far more questions. Strong partnerships among implementation researchers, practitioners, funding agencies, and behavioral health systems and providers will be essential to fully understand and address these complex problems. By collaborating and learning together, we can hope to realize widespread, sustainable EBP implementation at a level that can produce population-level benefits in behavioral health.
